# AI‐assisted warfarin dose optimisation with CURATE.AI for clinical impact: Retrospective data analysis

**DOI:** 10.1002/btm2.10757

**Published:** 2025-02-03

**Authors:** Tiffany Rui Xuan Gan, Lester W. J. Tan, Mathias Egermark, Anh T. L. Truong, Kirthika Kumar, Shi‐Bei Tan, Sarah Tang, Agata Blasiak, Boon Cher Goh, Kee Yuan Ngiam, Dean Ho

**Affiliations:** ^1^ Division of Surgery Ng Teng Fong General Hospital Singapore Singapore; ^2^ The N.1 Institute for Health (N.1), National University of Singapore Singapore Singapore; ^3^ The Institute for Digital Medicine (WisDM), National University of Singapore Singapore Singapore; ^4^ Department of Biomedical Engineering College of Design and Engineering, National University of Singapore Singapore Singapore; ^5^ Yong Loo Lin School of Medicine National University of Singapore; ^6^ Department of Pharmacology Yong Loo Lin School of Medicine, National University of Singapore Singapore Singapore; ^7^ Division of Haematology‐Oncology National University Hospital Singapore Singapore; ^8^ Division of Surgery National University Hospital Singapore Singapore; ^9^ The Bia‐Echo Asia Centre for Reproductive Longevity and Equality (ACRLE), National University of Singapore Singapore Singapore

**Keywords:** artificial intelligence, blood coagulation, clinical, decision support systems, precision medicine, warfarin

## Abstract

**Background:**

Standard‐of‐care for warfarin dose titration is conventionally based on physician‐guided drug dosing. This may lead to frequent deviations from target international normalized ratio (INR) due to inter‐ and intra‐patient variability and may potentially result in adverse events including recurrent thromboembolism and life‐threatening hemorrhage.

**Objectives:**

We aim to employ CURATE.AI, a small‐data, artificial intelligence‐derived platform that has been clinically validated in a range of indications, to optimize and guide warfarin dosing.

**Patients/methods:**

A personalized CURATE.AI response profile was generated using warfarin dose (inputs) and corresponding change in INR between two consecutive days (phenotypic outputs) and used to identify and recommend an optimal dose to achieve target treatment outcomes. CURATE.AI's predictive performance was then evaluated with a set of metrics that assessed both technical performance and clinical relevance.

**Results and conclusions:**

In this retrospective study of 127 patients, CURATE.AI fared better in terms of Percentage Absolute Prediction Error and Percentage Prediction Error of 20% compared to other models in the literature. It also had negligible underprediction bias, potentially translating into lower bleeding risk. Modeled potential time in therapeutic range with CURATE.AI was not significantly different from physician‐guided dosing, so it is on‐par yet provides a systematic approach to warfarin dosing, easing the mental‐burden on guesswork by physicians.

This study lays the groundwork for the prospective study of CURATE.AI as a clinical decision support system. CURATE.AI may facilitate the effective use of affordable warfarin with a well‐established safety profile, without the need for costly, new oral anticoagulants. This can have significant impact both on the individual and public health.

**Translational Impact Statement**

Physician‐guided warfarin dosing lead to frequent deviations from INR and adverse events. CURATE.AI, an artificial intelligence‐derived platform, has potential as a Clinician Decision Support System to improve accuracy of warfarin dosing decisions compared to physician‐guided dosing. This can facilitate the effective use of affordable warfarin with a well‐established safety profile, without the need for costly, new oral anticoagulants. This can have significant impact both on the individual and public health.

## 1 BACKGROUND

Warfarin is one of the most widely prescribed oral anticoagulant^1^, ^2^, ^3^ that is FDA‐approved for the prophylaxis and treatment of thromboembolic conditions, including deep venous thrombosis, pulmonary embolisms, atrial fibrillation, cardiac valve replacement and dilated cardiomyopathies.^4^, ^5^, ^6^ These conditions affect a large proportion of the population and contribute to significant morbidity and mortality.^7^ Pulmonary embolism alone results in 250,000 hospitalizations each year and approximately 200,000 deaths.^8^ Approximately 200,000 strokes each year can be attributed to thromboembolism.^9^ There are 10 million cases of venous thromboembolism occurring every year worldwide^10^, ^11^ and over 7 million cases of ischemic stroke.^12^

Preventing and treating thromboembolism is thus of major public health importance. Standard‐of‐care (SOC) for warfarin dose titration is conventionally based on physician‐guided drug dosing where physicians make educated guesses about factors causing deviation from the target range and the amount by which to adjust the dose in response.^3^ The complexity of inter‐ and intra‐patient variability in response to drug dosage lead to frequent deviations from the target international normalized ratio (INR) ranges.^13^ INR is a measure derived from prothrombin time that reflects the coagulability of blood. INR is recognized as the standard for laboratory monitoring of warfarin therapy as it is convenient to measure and is an established means for clinicians to monitor anticoagulation therapy and use for warfarin dose adjustment.^14^, ^15^, ^16^ Keeping a patient's INR within target therapeutic range is critical to the maintenance of anticoagulation in patients at risk of venous thromboembolism.^17^, ^18^ Given warfarin's narrow therapeutic index, over‐ and under‐coagulation may potentially result in adverse events including recurrent thromboembolism and life‐threatening hemorrhage.^19^, ^20^, ^21^ This forms a major challenge in warfarin therapy causing significant medical and economic consequences and diminishing the patient's quality of life.^22^

This has led to a recent shift toward the use of expensive New Oral Anticoagulants (NOACs) like Apixaban, Rivaroxaban, and Dabigatran.^23^ However, there remain indications and comorbidities for which warfarin is preferred, such as prosthetic valves,^24^, ^25^, ^26^, ^27^ antiphospholipid syndrome,^28^ and a high risk of gastrointestinal bleeding.^28^, ^29^, ^30^, ^31^ Furthermore, warfarin is cost‐effective, has a well‐established safety profile,^32^ can be managed by patients themselves with PT/INR self‐testing devices^33^ and can be easily reversed in case of over‐coagulation whereas, specific reversal agents for NOACs face issues of clinical availability and affordability.^34^ Warfarin thus maintains a stronghold in the armamentarium of anticoagulation.^35^

In an attempt to better manage warfarin therapy, various dosing algorithms have been developed.^36^, ^37^ However, these algorithms are not without limitations. First, they often require parameters ranging from environmental to genetic factors known to affect warfarin pharmacokinetics (PK) and pharmacodynamics (PD).^37^, ^38^, ^39^ The incorporation of such extensive clinical information makes them cumbersome and resource‐demanding. For example, dosing algorithms that require input of genetic information necessitates the availability of rapid‐turnaround genotyping which may not be feasible especially in resource‐poor environments.^7^ Second, most of them are population‐based and hence require population‐derived values or require training on extensive population data, with a limited ability to account for inter‐ and intra‐individual variability.^40^ There thus remains a need for a truly personalized yet implementable approach for managing warfarin dosing.

CURATE.AI is an artificial intelligence (AI)‐derived, small data platform that has been clinically validated for dose optimisation in oncology and immunosuppression, among other indication areas.^40^, ^41^, ^42^, ^43^, ^44^, ^45^, ^46^, ^47^, ^48^, ^49^ CURATE.AI follows a similar essence to the personalized construction and use of digital twins to potentially tailor treatments for patients.^50^ CURATE.AI correlates patient‐specific drug and dose (inputs) to the corresponding efficacy or toxicity (phenotypic outputs) to generate a N‐of‐1 (personalized) CURATE.AI response profile.^40^ Based on the profile, CURATE.AI identifies and recommends an optimal dose to achieve the target treatment outcomes for the individual patient whose data were provided. As more patient dose and response data are collected along the course of treatment, the CURATE.AI profile can evolve with the patient's response across dose range and time, resulting in a robust and sustainable dose optimisation process.^40^ Of note, population‐based big data sets are not required or utilized in CURATE.AI.^49^ Another key feature of CURATE.AI is the optimisation based on phenotypic output, rather than modeling the complex system that determines drug effects: drug bioavailability, PK/PD, drug–drug interactions, medication adherence or patient comorbidities among many others. In CURATE.AI, these phenomena are inherently incorporated into the input–output relationship and, as such, CURATE.AI is both disease mechanism‐independent and indication‐agnostic.^50^ Taken together, CURATE.AI is a promising algorithm for warfarin therapy.

In this study, we aimed to employ CURATE.AI to retrospectively model the optimisation of warfarin dosing for individual patients, based on each patient's data only. We explored both quadratic and linear relationships as the basis for CURATE.AI dose optimisation.^20^, ^40^ CURATE.AI's performance was then evaluated with a set of metrics that assessed both technical and clinical performance to demonstrate CURATE.AI's robustness and support the potential application of CURATE.AI as a clinical decision support system (CDSS) in the prospective management of warfarin dosing.

## 2 METHODS

### 2.1 Data collection

This retrospective study was approved by the National Healthcare Group Domain Specific Review Board, NHG DSRB 2020/00019. The study analyzed the clinical records of 127 patients who underwent warfarin therapy for deep venous thrombosis, pulmonary embolism and atrial fibrillation in the year of 2018 in the National University Hospital (NUH), a tertiary hospital in Singapore. De‐identified data containing patient demographics, prescribed warfarin doses, corresponding observed INR and date of INR measurements was obtained from Discovery.AI, a platform that collates NUH patient data in an anonymized fashion. Data on genetic factors were not required in the CURATE.AI dosing optimisation process and were not collected.

### 2.2 Personalized dosing optimisation process with CURATE.AI

The process of CURATE.AI dose optimisation using second order (CURATE.AI Quadratic) and first order functions (CURATE.AI Linear) is demonstrated below using simulated patient data.

#### 2.2.1 Simulated CURATE.AI quadratic process

**FIGURE 1:**
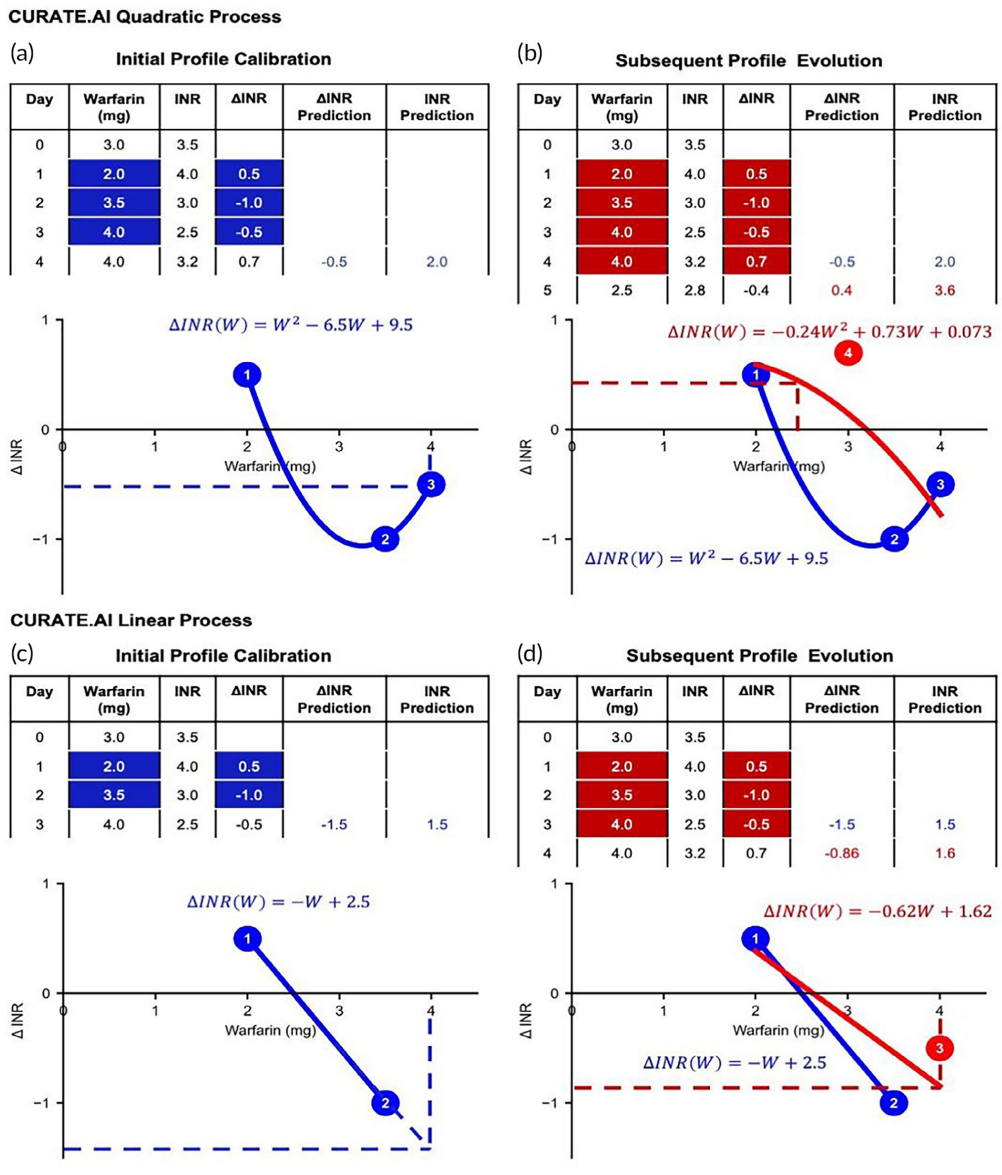
CURATE.AI quadratic and linear process with simulated data. Simulated records of patient's prescribed warfarin doses and their corresponding INR measurements were used. CURATE.AI Quadratic (a, b) The patient's initial profile (blue) was calibrated using dose–response data pairs on days 1 to 3. The profile was used to predict the patient's ∆INR and INR response to the warfarin dose given on day 4. Numbers within the circles correspond to the dosing days with the given warfarin dose. (a) Initial calibrated profile (blue) using the dose–response data pairs from days 1 to 3. (b) Assuming no systemic and regimen changes, the profile evolves to include the observed dose–response data pair on day 4. CURATE.AI Linear (c and d): The patient's initial profile (blue) was calibrated using dose response data pairs on days 1 and 2. The profile was used to predict the patient's ∆INR and INR response to the warfarin dose given on day 3. Numbers within the circles correspond to the dosing days with the given warfarin dose. (c) Initial calibrated profile (blue) using the dose–response data pairs from days 1 and 2. (d) With no systemic and regimen changes, the profile evolves to include the observed dose–response data pair on day 3.

Figure 1a,b demonstrate the CURATE.AI Quadratic dosing optimisation process using simulated data. In this simulation, warfarin dose was modulated against the change in the phenotypic output—change in INR between two consecutive days (∆ INR). Using a minimum of three uniquely modulated doses, the administered warfarin dose (input), represented by W, and the corresponding ∆ INR response (output) from days 1, 2, and 3 were mapped via a second order response equation $∆\mathrm{INR}\left(W\right)=W^{2}-6.5W+9.5$ (Figure 1a) where the equation coefficients [1; −0.65; 9.5] were specific to the simulated case at the given timepoint. This equation represents the patient's profile. The ∆ INR and INR predictions for day 4 are computed from this initial profile with the next recorded dose of 4 mg.

With no systemic or regimen changes, the observed dose–response data pair on day 4 was included into the patient's profile, represented by the evolved response equation $\mathrm{INR}\;\left(W\right)=-0.24W^{2}+0.73W+0.073$ (Figure 1b) with new coefficients [−0.24; 0.73; 0.073] specific to the simulated case at the new timepoint. Predictions for ∆ INR and INR for day 5 are computed and the process is repeated for the remaining timepoints.

#### 2.2.2 Simulated CURATE.AI linear process

The CURATE.AI Linear process is similar to that of CURATE.AI Quadratic. It requires a minimum of 2, instead of 3, uniquely modulated doses and the corresponding phenotypic output to calibrate a profile. The modulated warfarin doses and ∆ INR response from days 1 and 2 are mapped via a first order response equation represented by $∆\mathrm{INR}\left(W\right)=-W+2.5$ (Figure 1c). From this initial calibrated profile and the next recorded dose of 4 mg, the predictions for ∆ INR and INR for day 3 are computed.

With no systemic or regimen changes on day 3, the observed dose response data pair on day 3 is included in the profile, yielding a response equation $\mathrm{INR}\;\left(W\right)=-0.62W+1.62$ (Figure 1d). Predictions for ∆ INR and INR for day 4 are computed and the process repeats for the remaining timepoints.

### 2.3 Evaluation of CURATE.AI's predictive performance

In order to evaluate CURATE.AI's INR predictive performance, six metrics were identified: three technical performance metrics and three clinically relevant performance metrics (Table 1).

**TABLE 1:** Table of metrics.

Group	Metrics	Formula	Reference
Technical performance metrics	Percentage absolute prediction error (PAPE)	$\frac{\mathrm{abs}\left(\text{Predicted}\;\mathrm{INR}-\text{Observed}\;\mathrm{INR}\right)}{\text{Observed}\;\mathrm{INR}}\%$	Vadher et al.^51^
	Percentage prediction error (PPE)	$\frac{\text{Predicted}\;\mathrm{INR}-\text{Observed}\;\mathrm{INR}}{\text{Observed}\;\mathrm{INR}}\%$	Asiimwe et al.^37^
	PPE 20%	−20%<PPE<20%	Xue et al.^35^
Clinically relevant performance metrics	INR clinical prediction error (CPE)	Lower bound<PredictedINR−ObservedINR<Upper bound	Newly devised
	Underprediction bias	PredictedINR−ObservedINR<0	
	Modeled potential time in therapeutic range (TTR)	$\frac{\mathrm{No}.\;\text{of prediction events marked}\;\mathrm{as}\;\text{success}}{\text{Total}\;\mathrm{no}.\;\text{of predictions made}}\%$	

#### 2.3.1 Technical performance metrics

Three metrics were selected from literature review to perform cross model comparison for models used for serial INR predictions.^35^, ^37^, ^51^ At the time of analysis, there were only a limited number of models in the literature that used INR as the predicted response. These were shortlisted and compared against CURATE.AI using metrics reported in their respective analysis.

First, percentage absolute prediction error (PAPE) was selected from Vadher et al.'s evaluation of their population‐based pharmacokinetic/pharmacodynamic (PKPD) model. The model uses an empirical model which describes the relationship between vitamin K dependent clotting factors and INR, and the Bayesian method for parameter estimation.^51^ CURATE.AI's PAPE was compared with Vadher's Bayesian PKPD model^42^ by identifying patients with sufficient data for at least four predictions to match the number of predictions between the two models. Statistical comparison between the PAPE for CURATE.AI and Vadher's model was not able to be performed because of the limited retrospective data set in this study and the lack of availability of raw data from these models in the literature.

Second, percentage prediction error (PPE) was chosen to benchmark CURATE.AI's performance against the performance of Xue et al.'s dose–response model, Xue et al.'s PKPD model and Hamberg et al.'s PKPD model reported for an independent dataset. The mean/median percentage PE for those models was obtained by summing each category across the five hospitals' data they were externally evaluated against.^2^, ^35^ Of note, all those three models require population‐based estimates of parameters alongside patient specific parameters like genetic makeup, observed warfarin dose and INR response, whereas CURATE.AI only requires 2 to 3 longitudinal dose–response data pairs of the treated patient to generate its first recommendation.

Lastly, INR PPE of 20% (PPE 20%) was selected from Xue et al.'s model evaluation.^35^ PPE 20% considers INR prediction error within ±20% as ideal. INR predictions with an error <−20% and >20% are considered as underprediction and overprediction, respectively. The authors did not provide substantiated reasoning behind the choice of 20% as an acceptable threshold but it forms a useful technical comparison metric to compare the predictive performance of CURATE.AI with other models in the literature. CURATE.AI's results were only compared against Xue et al.'s findings with respect to this metric due to the absence of relevant data required to compare against Vadher et al.'s model.

#### 2.3.2 Clinically relevant performance metrics

Three metrics were newly devised to assess the clinical relevance of CURATE.AI (Table 1). A literature review revealed a lack of clinically relevant metrics to evaluate serial INR prediction performance.^34^ With the goal of CURATE.AI developed as a CDSS in the prospective management of warfarin dosing, it is essential that clinically relevant metrics are part of the criteria for performance evaluation.

First, INR clinical prediction error (CPE), assesses the percentage of the predictions whose CPE was within prespecified, clinically motivated error threshold. INR therapeutic range for most patients is 2.0–3.0. With the risk of bleeding increases significantly with INR > 5.0 and risk of thromboembolism with INR < 1.5,^14^, ^15^, ^16^, ^52^ we considered 2.0 and 0.5 error thresholds as clinically relevant and included them in the CURATE.AI performance assessment.

Second, underprediction bias, focuses on negative prediction error. Underprediction bias increases the risk of overdosing warfarin, in turn increasing the patient's risk of bleeding that is not easily reversed.

**FIGURE 2:**
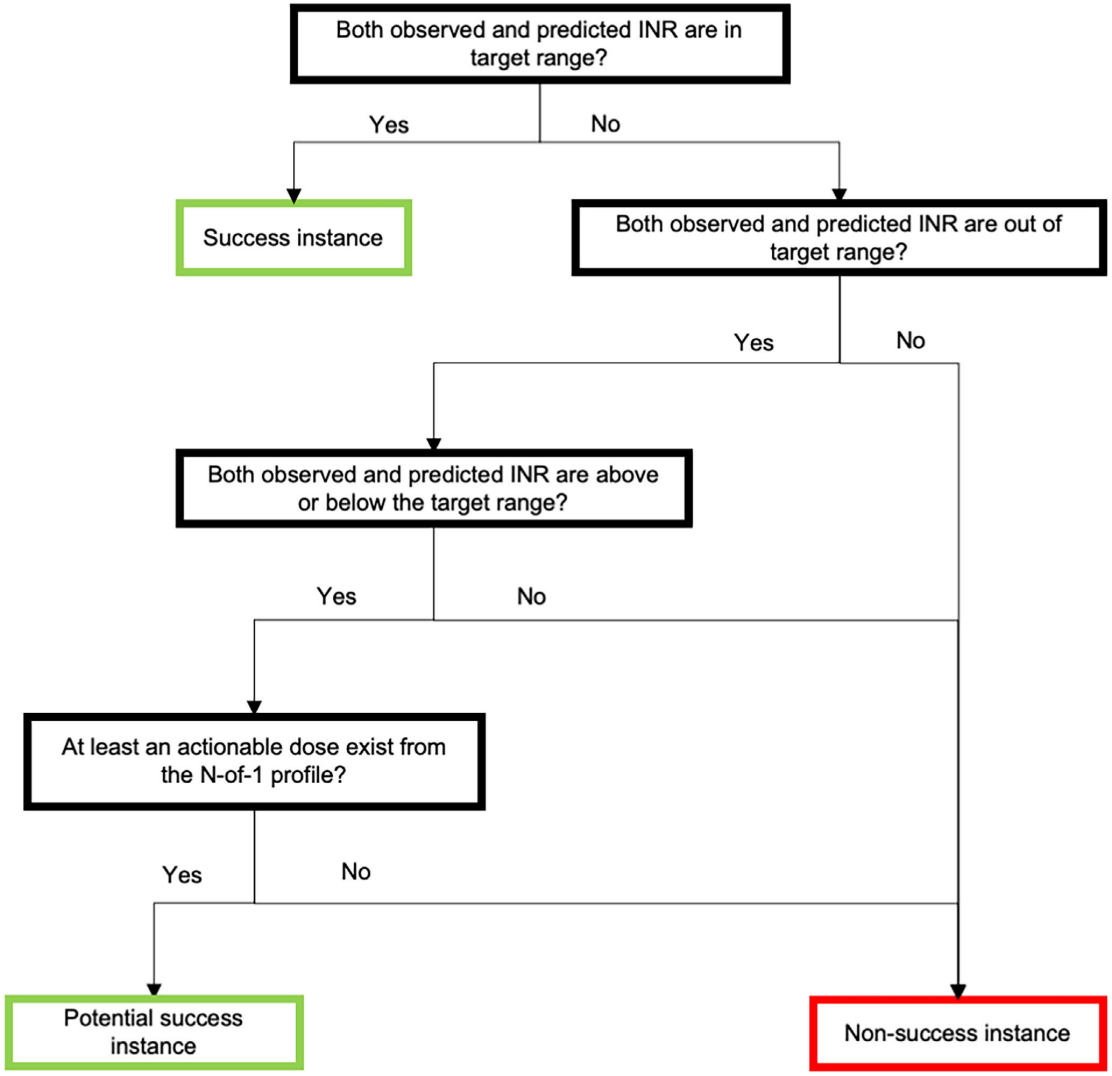
Decision flowchart of modeled potential TTR. Each prediction instance is first determined if they count as a success instance using an algorithm.

Lastly, modeled potential time in therapeutic range (TTR) was generated to estimate CURATE.AI's capability to support warfarin dosing in prolonging INR within the therapeutic range. Each prediction instance was labeled as success or non‐success (Figure 2). To be labeled as success—leading to INR within therapeutic range—the dosing instance had to fulfill the following:

1. Both the corresponding predicted INR and the observed INR were in therapeutic range—CURATE.AI correctly predicted the INR to be in target range
2. Predicted INR and the observed INR were both below or both above the therapeutic range but there exist an actionable dose—a dose that CURATE.AI would have potentially recommended that lies within 0 to 14 mg and is not the observed dose, that would achieve target INR.

The CURATE.AI modeled potential TTR for a single patient was then calculated by the percentage of success instances over the total number of predictions. This modeled potential TTR was compared against physician‐guided TTR which was calculated by the percentage of prediction events resulting in an INR in the therapeutic range over the total number of INR measurements, beginning from the first CURATE.AI prediction.

### 2.4 Statistical analysis

Data analysis was performed using scripts written in Python (Version 3.11.0). Normality test was performed with Shapiro–Wilk test at α = 0.05. Statistical comparison were performed with Wilcoxon signed‐rank test at α = 0.05.

## 3 RESULTS

### 3.1 Patient population

The study analyzed the clinical records of 127 patients who underwent warfarin therapy for deep venous thrombosis, pulmonary embolism and atrial fibrillation in the year of 2018 in the National University Hospital (NUH). The demographic characteristics of these patients are listed in Table 2.

**TABLE 2:** Patient demographics.

Variables	Patients (*N* = 127)
Sex	
Male	76 (59.8%)
Female	51 (40.2%)
Age [years]	58.5 [18–99]^a^
Ethnicity	
Chinese	83
Malay	18
Indian	10
Others	16

^a^ Mean (range).

**FIGURE 3:**
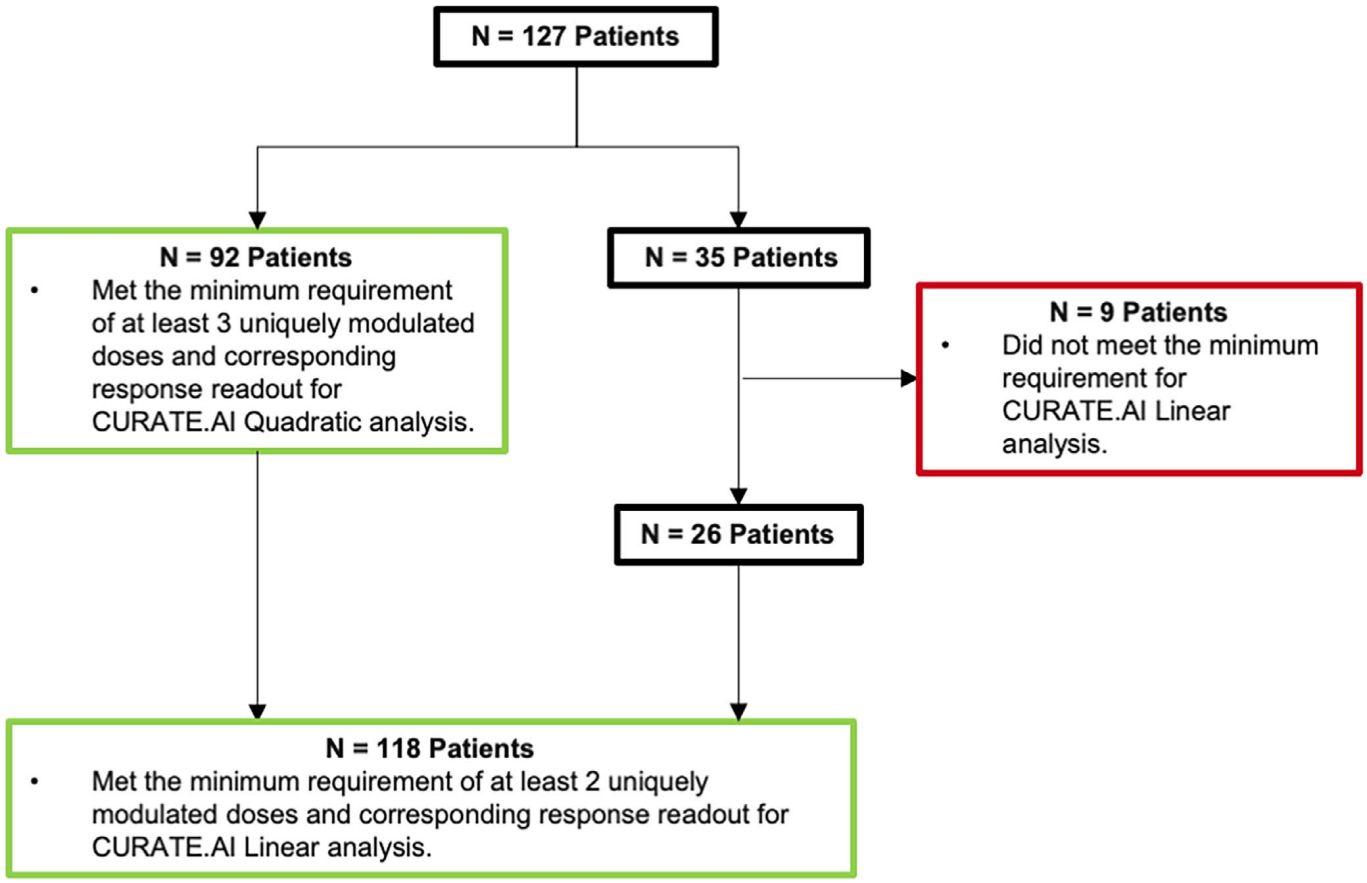
Internal validation data screening flow. A total of *N* = 127 de‐identified patient data were retrieved from the Discovery.AI Platform. *N* = 92 and *N* = 118 patient data met the requirements of a minimum of 3 and 2 modulated doses and corresponding response readout for CURATE.AI Quadratic and CURATE.AI Linear profile analysis, respectively. *N* = 9 patient data did not meet the requirement and were excluded from the analysis. *N* = number of patients.

Patients with sufficient dose–response data to generate at least one second‐order dose–response profile and were able to generate a minimum of one INR prediction were identified. A total of *N* = 92 patients and *N* = 118 patients were identified to have met the requirement for CURATE.AI Quadratic and CURATE.AI Linear analysis, respectively (Figure 3). The target range of INR was assumed 2–3 for all patients.

### 3.2 Out‐of‐bounds predictions

It is possible for models to generate predictions outside of physiological and clinical quantitation means. Out‐of‐Bounds (OOB) predictions refer to INR predictions that are impossible in the physiological context (negative INR) and values beyond the limit of quantitation (INR > 15).^53^ As CURATE.AI is intended as CDSS with physician oversight, the OOB predictions will be actively managed should they occur. 1.15% (*n* = 9 out of 786 predictions) of INR predictions from CURATE.AI Quadratic and 0.88% (*n* = 9 out of 1025 predictions) of INR predictions from CURATE.AI Linear model were considered OOB and removed from the subsequent analyses.

### 3.3 INR distribution

**FIGURE 4:**
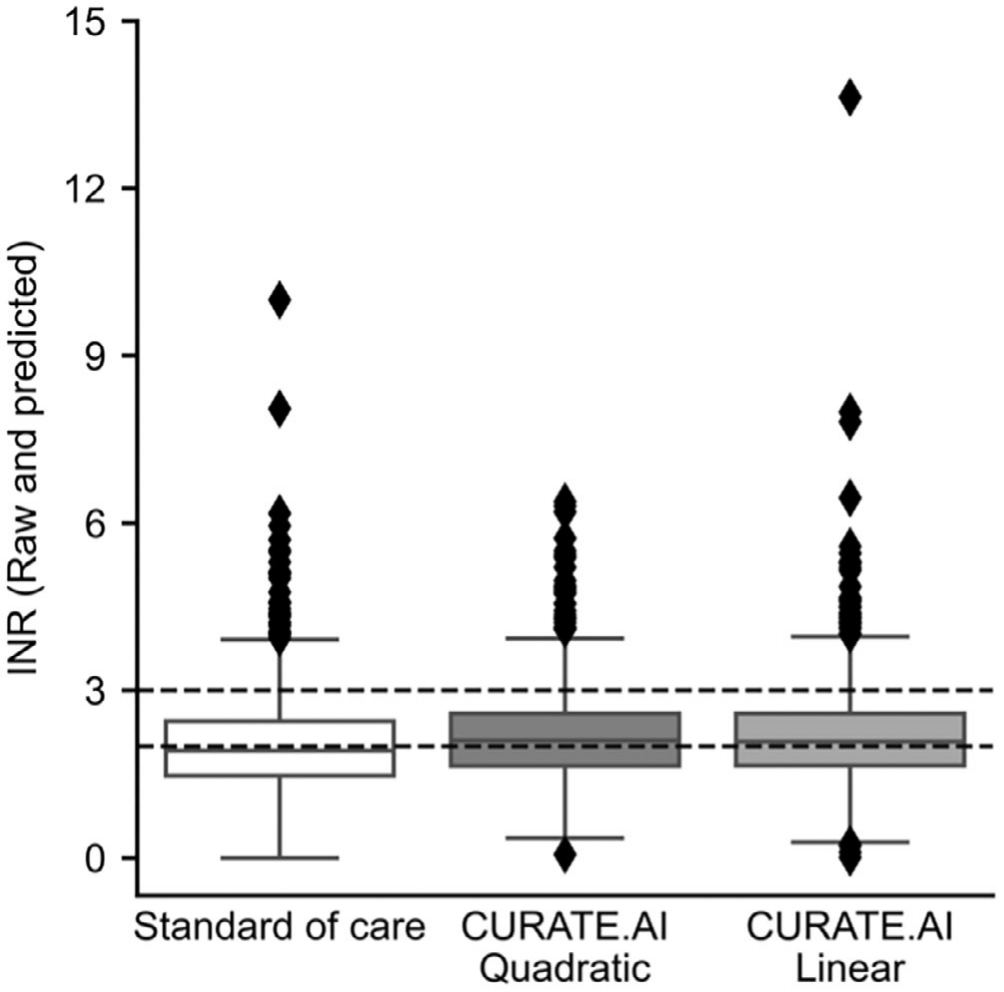
INR distribution. Raw and predicted INR boxplots for INR measured in patients in standard of care (*N* = 118, *n* = 1507) and predicted using CURATE.AI Quadratic (*N* = 92, *n* = 777) and CURATE.AI Linear (*N* = 118, *n* = 1016). Interquartile ranges are 0.98, 0.95, and 0.94 for standard of care, CURATE.AI Quadratic and CURATE.AI Linear, respectively. The lower bar and upper bar indicate median ± 1.5*IQR. Dashed lines represent the assumed 2–3 therapeutic range. *N* = number of patients, *n* = total number of measured events or predicted events.

The boxplots of INR values of the dataset, together with the predicted INR values using CURATE.AI Quadratic and CURATE.AI Linear are shown in Figure 4. The distribution of INR values that are within and out of the assumed therapeutic range of INR 2–3 are shown in Table 3.

**TABLE 3:** INR distribution (number [%]).

Data/Model	INR < 2	Therapeutic INR	INR > 3
Standard of care (*n* = 1507)	810 (53.7%)	559 (37.1%)	138 (9.16%)
CURATE.AI Quadratic (*n* = 777)	340 (43.8%)	335 (43.1%)	102 (13.1%)
CURATE.AI Linear (*n* = 1016)	451 (44.4%)	443 (43.6%)	122 (12%)

*Note*: Counts of observed and predicted INR from the dataset and CURATE.AI, respectively. n: number of measured events or predicted events.

### 3.4 CURATE.AI technical performance metrics

#### 3.4.1 Percentage absolute prediction error

The median Percentage absolute prediction error (PAPE) across all predictions for CURATE.AI Quadratic and CURATE.AI Linear are shown in Table 4.

**TABLE 4:** CURATE.AI PAPE.

Model	PAPE
	Median [95% CI]
CURATE.AI quadratic (*n* = 777)	14.9 [13.7–17.0]
CURATE.AI linear (*n* = 1016)	14.3 [13.3–15.5]

*Note*: Values are reported as median [95% CI]. *n*: total number of prediction events.

To compare CURATE.AI's performance with Vadher's Bayesian PKPD model, patients with sufficient data for at least four predictions were identified for this analysis (CURATE.AI Quadratic: *N* = 62, CURATE.AI Linear: *N* = 75).

Overall trend in median PAPE for both CURATE.AI Quadratic and CURATE.AI Linear were similar with Vadher's Bayesian PKPD model—the median PAPE was worse after the first prediction, which subsequently gets better.

**FIGURE 5:**
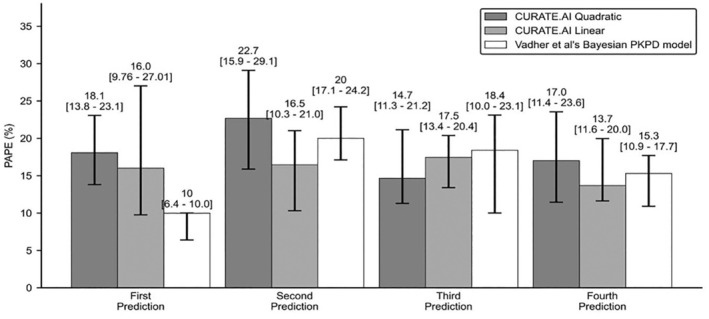
Serial Percentage Absolute Prediction Error (PAPE). Median PAPE for the first four serial INR predictions for CURATE.AI Quadratic (*N* = 62) CURATE.AI Linear (*N* = 75) and Vadher et al.'s Bayesian PKPD model (*N* = 74). The error bar represents 95% confidence interval. Comparison rests on the assumption that the underlying samples validated for each model are representative of a sample of patient population. *N* = number of patients.

However, for Vadher et al.'s model a two‐fold worsening in PAPE was observed after the first prediction. Each day‐to‐day differences in PAPE for CURATE.AI Quadratic and CURATE.AI Linear on the other hand were not statistically significant (Figure 5).

The median PAPE for CURATE.AI Quadratic and Linear were similar (Quadratic: 17.0%, Linear: 13.7%) with Vadher et al.'s model (15.3%) for the fourth prediction when the Vadher et al.'s model was expected to perform the best.

#### 3.4.2 PPE and PPE 20%

The median PPE across all predictions generated by CURATE.AI Quadratic (*n* = 777) and CURATE.AI Linear (*n* = 1016) was 1.2 and 1.3, with the 95% confidence interval [−0.6 to 2.6] and [−0.2–2.9], respectively.

**FIGURE 6:**
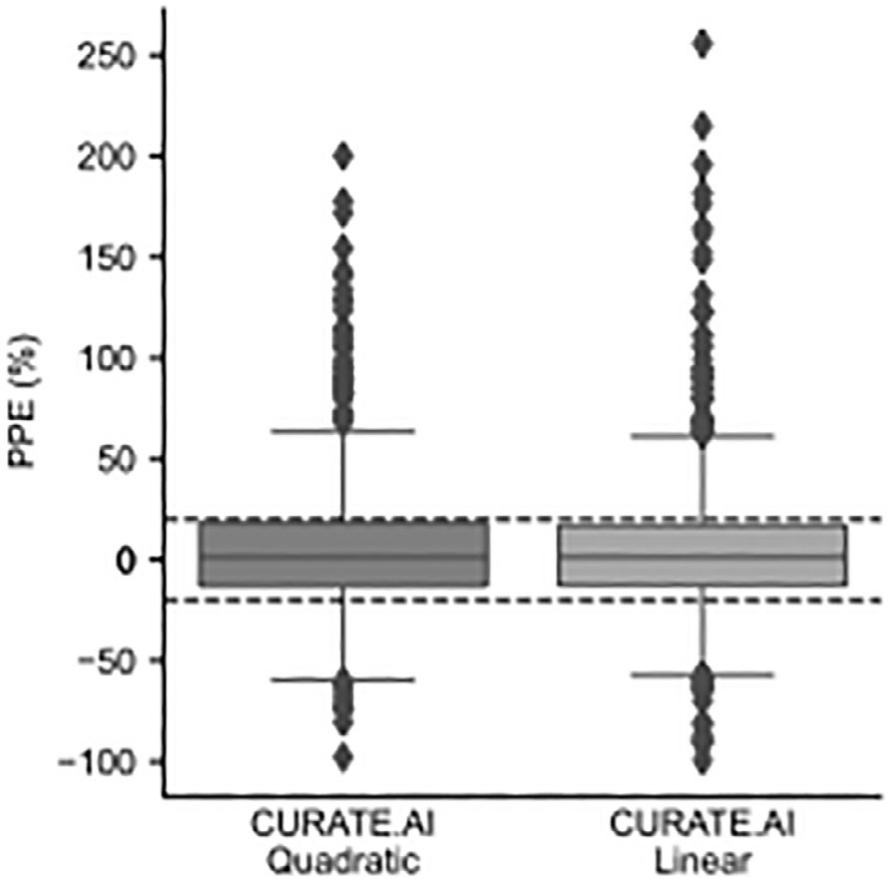
Percentage prediction error (PPE). INR PPE for both CURATE.AI quadratic (*n* = 777) and linear (*n* = 1016). Interquartile ranges (IQR) are 31.2 and 29.7 for CURATE.AI quadratic and linear, respectively. The lower bar and upper bar indicate median ± 1.5*IQR. Dashed lines represent the ±20% threshold for ideal predictions. *n* = total number of prediction events.

The PPE 20% results for CURATE.AI Quadratic and Linear are shown in Table 4 and the distribution box plots are shown in Figure 6. 61.0% (*n* = 474) and 62.3% (*n* = 633) of INR predictions generated by CURATE.AI Quadratic and CURATE.AI Linear, respectively, were considered ideal (INR prediction within ±20%). Both CURATE.AI models fared better in their predictive performance compared to Xue et al.'s dose–response model (57.9% ideal INR predictions), Xue et al.'s PKPD model (29.0% ideal INR predictions) and Hamberg et al.'s PKPD model (50.7% ideal INR predictions) (Table 5).

**TABLE 5:** INR PPE 20% from CURATE.AI and literature (number (percentage)).

Model	Under prediction	Ideal prediction	Over prediction
CURATE.AI Quadratic (*n* = 777)	122 (15.7%)	474 (61.0%)	181 (23.3%)
CURATE.AI Linear (*n* = 1016)	156 (15.4%)	633 (62.3%)	227 (22.3%)
Xue et al. Xue et al. Dose–response model^32^	532 (20.2%)	1525 (57.9%)	576 (21.9%)
Xue et al. PKPD model^51^	1390 (52.8%)	764 (29.0%)	479 (18.2%)
Hamberg et al. PKPD model^2^	714 (27.1%)	1336 (50.7%)	583 (22.1%)

*Note*: Categories (under prediction, ideal prediction and over prediction) are based on a 20% threshold determined from literature. Comparisons between CURATE.AI and Xue et al.'s dose–response model, PKPD model and Hamberg et al.'s PKPD model rests on the assumption that the underlying samples validated for each model are representative of a sample of patient population. *n*: total number of prediction events.

### 3.5 CURATE.AI clinically relevant performance metrics

#### 3.5.1 Clinical prediction error

**FIGURE 7:**
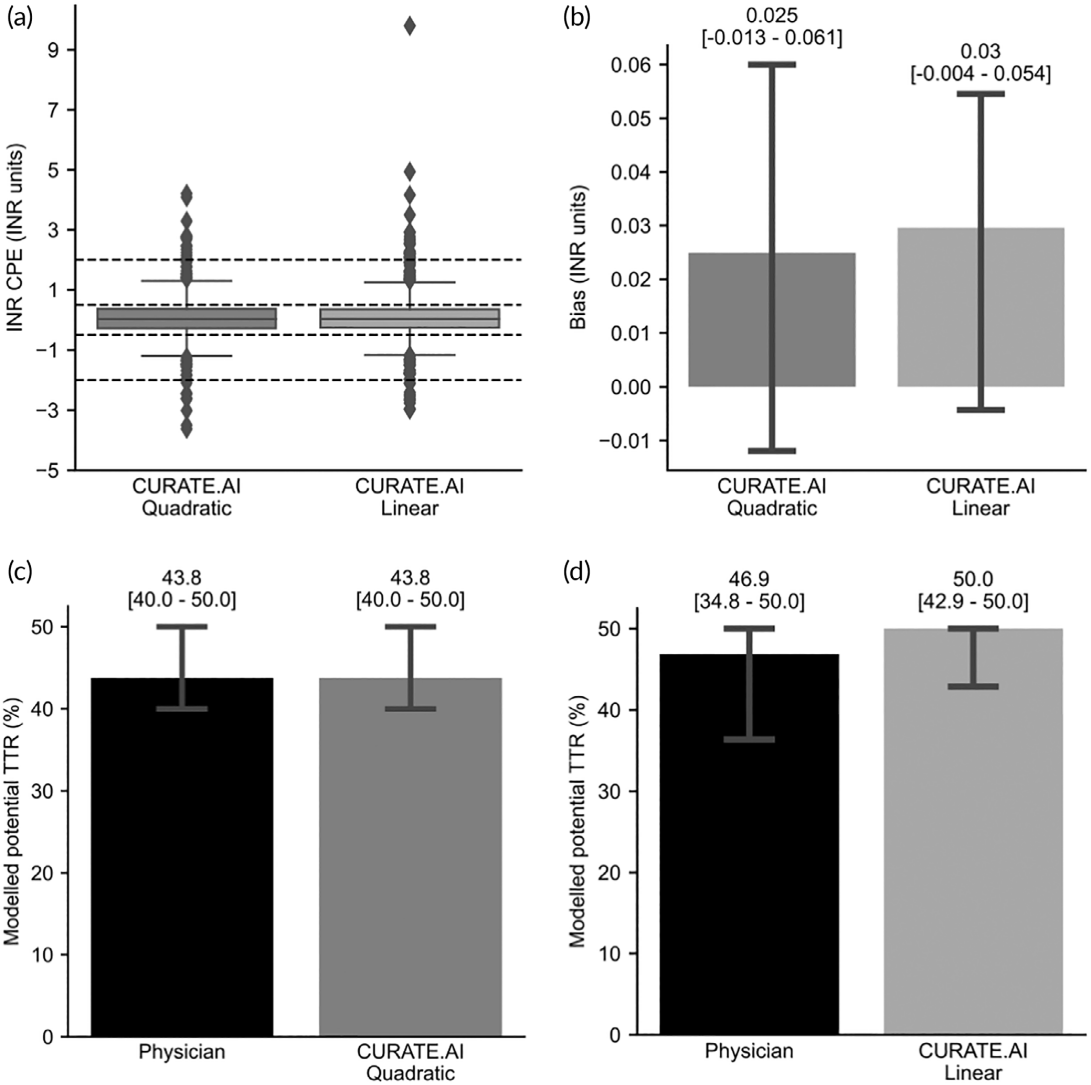
(a) INR clinical prediction error (CPE). CPE, in terms of INR units for both CURATE.AI quadratic (*n* = 614) and CURATE.AI linear (*n* = 774). Interquartile ranges (IQR) are 0.64 and 0.61 for CURATE.AI quadratic and linear, respectively. The lower bar and upper bar indicate median ± 1.5*IQR. Dashed lines represent the ±2.0 and ±0.5 threshold for ideal predictions. (b) Bias (INR units). Bias, expressed as prediction error for CURATE.AI quadratic (*n* = 614) and CURATE.AI linear (*n* = 774). The lower bar and upper bar represent the lower and upper range of 95% CI, respectively. (c, d) Modeled potential TTR (%). Modeled potential TTR expressed as percentage, for both (c) CURATE.AI quadratic (*N* = 91) and (d) CURATE.AI linear (*N* = 118) against physician‐guided dosing. The error bar represents the lower and upper range of 95% confidence interval, respectively. Normality testing performed using Shapiro–Wilk at 0.05 level of significance. Statistical comparison was performed with Wilcoxon signed rank test at 0.05 level of significance. *N* = number of patients. *n* = total number of prediction events.

The Clinical prediction error (CPE) results for CURATE.AI are shown in Table 5. For the CPE threshold of 2.0, 97.0% (*n* = 754) and 96.7% (*n* = 982) of predictions from CURATE.AI Quadratic and Linear were considered ideal, respectively. For the CPE threshold of 0.5, 66.3% (*n* = 515) and 68.0% (*n* = 691) of the predictions were considered ideal (Table 6 and Figure 7a).

**TABLE 6:** Clinical prediction error from internal evaluation of CURATE.AI (Number (Percentage)).

Threshold	Model	Under prediction	Ideal prediction	Over prediction
2.0	CURATE.AI Quadratic (*n* = 777)	8 (1.03%)	754 (97.0%)	15 (1.93%)
	CURATE.AI Linear (*n* = 1016)	11 (1.08%)	982 (96.7%)	23 (2.26%)
0.5	CURATE.AI Quadratic (*n* = 777)	114 (14.7%)	515 (66.3%)	148 (19.0%)
	CURATE.AI Linear (*n* = 1016)	142 (14.0%)	691 (68.0%)	183 (18.0%)

*Note*: Categories (under prediction, ideal prediction and over prediction) are based on 2.0 and 0.5 unit threshold. *n*: total number of prediction events.

#### 3.5.2 Underprediction bias

The bias for both CURATE.AI Quadratic and CURATE.AI Linear are shown in Figure 7b. The median bias for both CURATE.AI Quadratic and CURATE.AI Linear were 0.025 and 0.03, respectively, and the upper and lower bound values for the 95% confidence interval were clinically insignificant. This suggests that CURATE.AI predictions have negligible underprediction bias.

#### 3.5.3 Modeled potential time in therapeutic range (TTR)

The bar plots of modeled potential TTR for both CURATE.AI Quadratic and CURATE.AI Linear against physician‐guided dosing are shown in Figure 7c,d. Differences in modeled potential TTR between CURATE.AI and physician‐guided dosing were not statistically significant.

### 3.6 Comparison Between CURATE.AI Quadratic and CURATE.AI Linear

**FIGURE 8:**
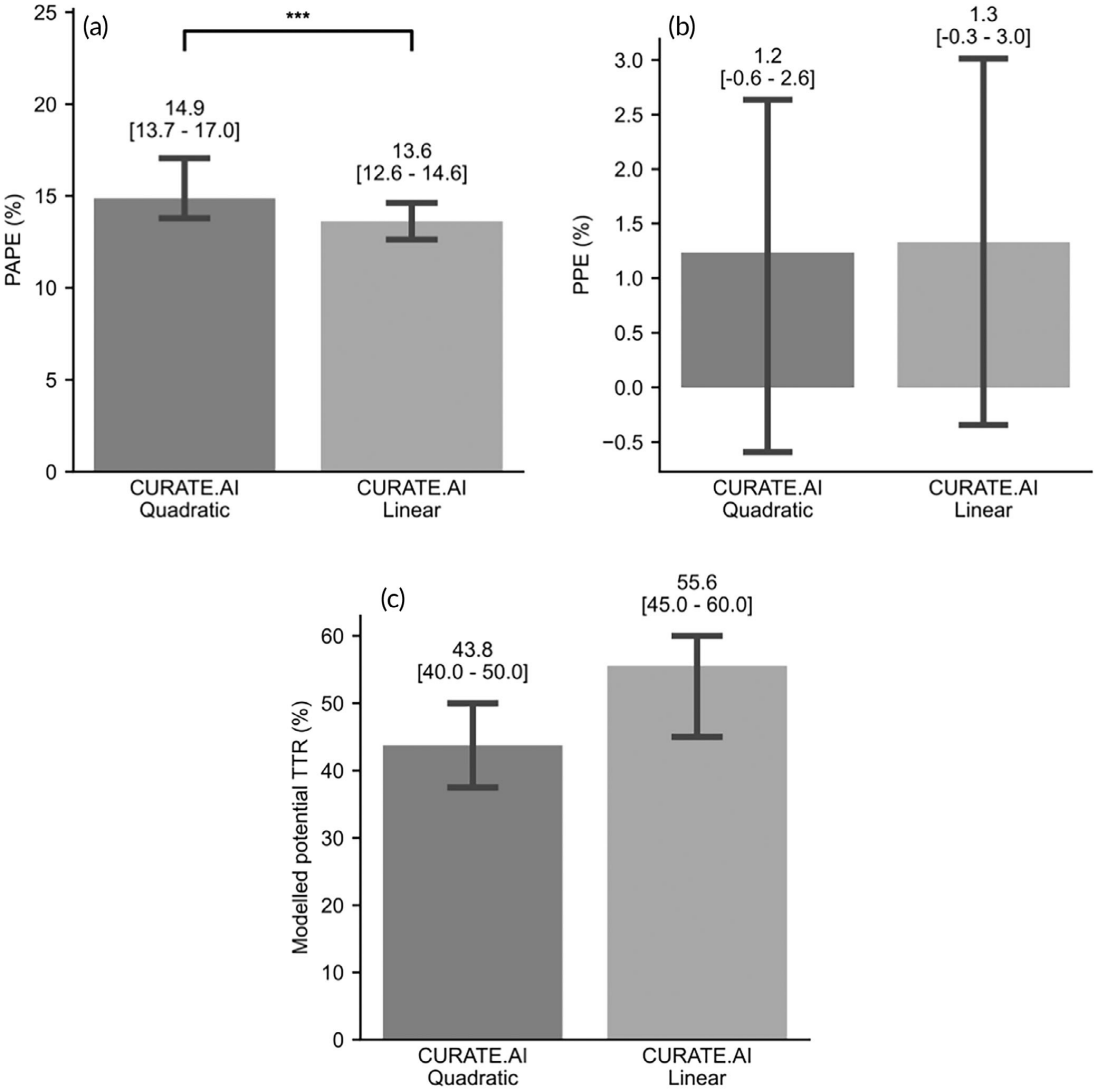
Bar plots comparing between CURATE.AI quadratic and linear in terms of precision, bias and modeled potential TTR (%). (a) PAPE for both CURATE.AI quadratic and linear (*n* = 777). (b) PPE for both CURATE.AI quadratic and linear (*n* = 777). (c) Modeled potential TTR (%) for CURATE.AI Quadratic and Linear (*N* = 91). The lower bar and upper error bar represents 95% confidence interval. Comparison rests on the assumption that the underlying samples validated for each model are representative of a sample of patient population. No statistically significant difference was detected between the conditions *t* with Wilcoxon signed rank test at α = 0.05. *N* = number of patients. *n* = total number of prediction events.

For additional evaluation for the more appropriate CURATE.AI model, pairwise comparison between CURATE.AI Quadratic and CURATE.AI Linear for the same prediction events (*n* = 777) across both technical performance and clinical relevance was performed and the results are shown in Figure 8. CURATE.AI Linear's predictions are significantly more accurate compared to CURATE.AI Quadratic (*p* < 0.001) (Figure 8a). No differences were observed for bias metrics percentage PE and modeled potential TTR (Figure 8b,c).

#### 3.6.1 Five‐fold cross validation

The five‐fold cross validation Median PAPE for both the training set and test sets were similar at 15.19 ± 0.46 and 15.49 ± 1.33, respectively, for CURATE.AI Quadratic; and 14.3 ± 0.16 and 14.62 ± 0.74, respectively, for CURATE.AI Linear. This suggests that factors such as patient demographics, clinical characteristics and genetic factors present in the used data set do not affect the predictive performance of both CURATE.AI Quadratic and CURATE.AI Linear and that the CURATE.AI models may be generalizable to other data sets of shared characteristics.

## 4 DISCUSSION

CURATE.AI successfully identified personalized dose–response relationships between warfarin and INR in 92 patients using CURATE.AI Quadratic and 118 patients using CURATE.AI Linear. CURATE.AI's robustness and potential application as a CDSS was then demonstrated with six metrics that assessed both technical performance and clinical relevance.

In comparison with existing models developed for INR response prediction, our models achieved predictions with precision considered as ideal (PPE <20%) more frequently as compared to Xue et al.'s dose–response model,^32^ Xue et al.'s PKPD model^54^ and Hamberg et al.'s PKPD model^2^ (Table 4). CURATE.AI Quadratic and CURATE.AI Linear demonstrated comparable technical performance to Vadher et al.'s model.^51^ CURATE.AI.'s median PAPE was within ±1.7% difference from Vadher et al.'s model by the fourth prediction.

Furthermore, there were fluctuations in median PAPE for Vadher's Bayesian PKPD model, notably after the first prediction. Although predictions improved in subsequent predictions, CURATE.AI's performance was consistent along the treatment timeline (Figure 5). This consistent performance despite common environmental fluctuations from day‐to‐day may be explained by the personalized approach of CURATE.AI that focuses on using the individual patient's data only, and its dynamic ability to evolve the generated profile by incorporating newly observed dose–response data pairs. This aligns with the hypothesis by Vadher et al that attributed their worsening performance over time to model misspecification^51^ which may result from the use of population‐based parameter estimates that may not be applicable to every patient.

In addition, this study highlights the utility of a small data approach in facilitating a more robust implementation of CDSS into a routine clinic use. Models that require more parameters or require training on large datasets suffer a trade‐off between comprehensive modeling and practicality for clinical deployment.^55^ While a comprehensive modeling approach that accounts for many predictors may demonstrate high predictive performance, it may be challenging for implementation as a clinical solution considering that large amounts of resources are required to obtain information on the predictors from every patient and to set up and/or train the model. Conversely, CURATE.AI achieved potentially superior performance while requiring significantly fewer parameters as algorithm inputs, making it attractive for deployment into a clinical practice.

Besides exploring the conventional second‐order polynomial (CURATE.AI Quadratic) as the backbone of CURATE.AI response predictions this analysis also explored the first‐order variant—CURATE.AI Linear. CURATE.AI Linear had comparable bias and superior precision to CURATE.AI Quadratic, albeit a slightly worse modeled potential TTR. With a potential to translate into reaching the optimal INR range sooner, as it requires one less temporal data pair, CURATE.AI Linear can potentially reduce clinic visits, blood tests and warfarin related emergency room visits without compromising on performance, thereby easing the burden on healthcare resources and improving the quality of life of patients.^22^

### 4.1 Importance of defining clinically relevant performance metrics for AI model evaluation

In this study, metrics were also newly devised based on both clinical experience and literature review. In the literature, only 7% of developed algorithms were assessed for clinical relevance at least once^37^ despite an increasing interest in bridging the gap between healthcare and artificial intelligence solutions for the development of clinically relevant artificial intelligence platforms.^56^ Given that the goal of CURATE.AI is to be applied as CDSS, it was essential to not just evaluate CURATE.AI's performance on technical aspects but also on clinical relevance.

CURATE.AI showed good performance in terms of all clinically relevant performance metrics. INR CPE demonstrated CURATE.AI being able to generate predictions considered ideal for nearly 97% and 70% of prediction events with the thresholds of 2.0 and 0.5, respectively. This is the first study to use INR CPE as an evaluation metric and future studies and clinical practice are needed to validate the ideal INR prediction error threshold. In the evaluation of the prediction bias, both CURATE.AI Quadratic and CURATE.AI Linear demonstrated negligible underprediction bias, translating to a lower risk of overdosing warfarin and warfarin‐induced bleeding. This metric was chosen because clinical tolerance for warfarin‐induced bleeding is lower than thromboembolism and more emphasis should thus be placed on minimizing warfarin overdose. Bleeding is the most common side effect of warfarin and occurs in up to 41% of patients,^7^, ^57^, ^58^ with the frequency of warfarin‐induced bleeding reported at 15% to 20% per year and severe bleeding episodes ranging between 1% and 3%.^59^, ^60^ While reversal agents for warfarin‐induced bleeding are available, there exist no optimal therapy for anticoagulant overdose, especially in emergency settings where time is of essence.^61^ Even minor bleeding can lead to withdrawal of therapy, thus depriving patients of the most effective, and often only, therapy available to prevent thromboembolism and leads to repeated office and sometimes emergency room visits.^7^ Conversely, risk of thromboembolism with patients on oral anticoagulants is relatively less significant. This is illustrated in a prospective, observational cohort study on 1024 patients receiving long term anticoagulation who experienced episodes of warfarin therapy interruptions reported an associated low risk of thromboembolism when interruptions are short (≤5 days).^62^

TTR is the most widely accepted method for determining the quality of warfarin therapy^11^, ^16^, ^63^, ^64^ defined as the percentage of time that patients' INR was within target range. Of the three commonly accepted methods for calculating TTR, the Rosendaal method measures the duration that a patient remains in therapeutic range,^65^ while the traditional method measures the ratio of measured therapeutic events over all measured events.^66^ Without running a prospective study on CURATE.AI in anticoagulation therapy, a TTR for CURATE.AI cannot be determined. Thus, the modeled potential TTR approach was devised as a proxy measure to provide a preliminary understanding on clinical efficacy using retrospective data. Enhancing time in therapeutic range is a critical determinant to maximizing efficacy and safety of warfarin^63^, ^67^, ^68^, ^69^, ^70^ because one of the major challenges in warfarin therapy is its narrow therapeutic index and the large inter‐individual variability in patient response to the drug,^7^ leading to fluctuations outside the therapeutic range and adverse events such as bleeding or recurrent thromboembolism.^67^, ^71^, ^72^

Results from the ROCKET AF trial showed that Sweden had the highest mean overall TTR at 75% compared to 64% in Singapore.^73^ A possible explanation would be the use of a computer based system with dosing algorithm—Sweden's national quality register for atrial fibrillation and oral anticoagulation (AuriculA) which has been shown to improve TTR compared to manual dosing.^74^ Similar to CURATE.AI, AuriculA provides a clinical decision tool aiding warfarin dosing using an algorithm requiring the patient's last two INR results and suggest the dosage of warfarin that can be accepted or manually changed.^75^ The results from this study saw no significant differences in modeled potential TTR between CURATE.AI and physician‐guided dosing. This could mean that CURATE.AI is an algorithm that is on‐par with physician‐guided dosing and yet provides a systematic approach to warfarin dosing which would ease the mental‐burden on guesswork by physicians.

### 4.2 CURATE.AI in a clinical workflow

CURATE.AI is envisioned as a CDSS that aims to make personalized dosing recommendations to the treating physician using its novel small‐data, personalized approach.

**FIGURE 9:**
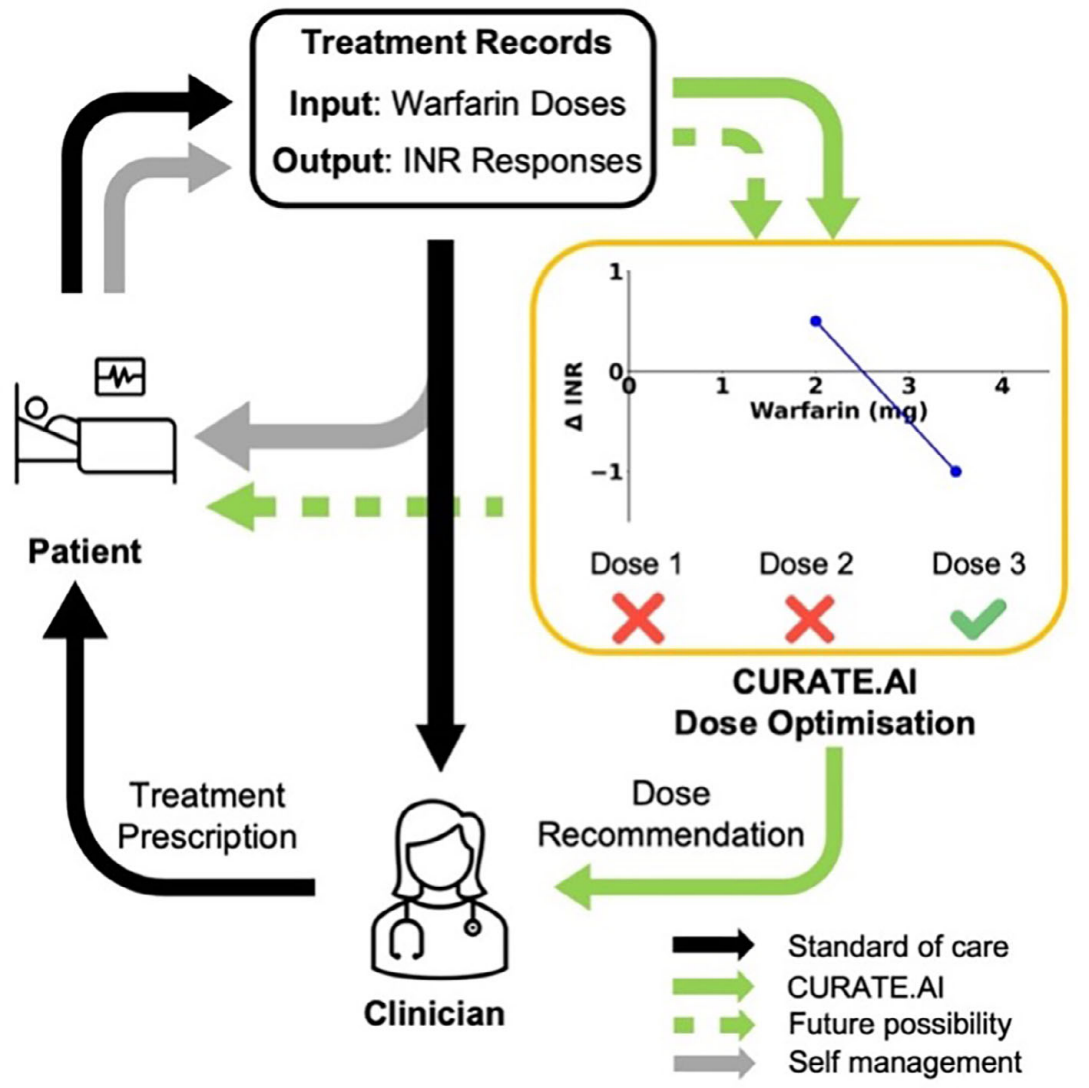
Proposed CURATE.AI Integration into a Clinical Workflow. Standard of care: Warfarin dose and corresponding INR response for a patient on anticoagulation therapy are charted and dose decision by the clinician is heavily relied on clinical experience. CURATE.AI: CURATE.AI maps the warfarin (inputs) dose and corresponding INR response (outputs) for that individual to calibrate a personalized dose–response profile. From this profile, CURATE.AI will recommend optimal doses to achieve the target INR to the clinician and, as a future possibility, potentially to patients for self‐management of oral anticoagulation.

Since warfarin dose titration already involves regular INR testing and dose adjustments with physician oversight, CURATE.AI can be easily integrated into the standard clinical workflow. With self‐monitoring of oral anticoagulation gaining traction,^76^ there is also a potential for stable patients to utilize CURATE.AI for self‐management of oral anticoagulation with suitable healthcare support as backup (Figure 9). In addition, CURATE.AI has the ability to dynamically evolve with the patient's status as newly measured dose–response data pairs are included into the profile, ensuring up‐to‐date recommendations with the latest patient status.

### 4.3 Limitations and future directions

The primary objective of this study was to test the potential of applying CURATE.AI in personalized dosing optimisation in anticoagulation therapy with warfarin. The optimisation analysis conducted in this study resulted in favorable prediction outcomes that support further prospective validation. However, this study is not without limitations. The analysis used dose and INR as parameters and INR has it's intrinsic limitations in being correlated with exact plasma warfarin concentrations as it may not detect the factors causing fluctuations in plasma warfarin concentrations immediately but INR is used as it is the most clinically relevant and established means of measuring the dose–response effect of warfarin.^14^, ^15^, ^16^, ^64^ The analysis broadly assumed an INR target range of 2–3 for all patients, while some patients might have a different INR target range which would have influenced the results of the modeled potential TTR. In addition, because of the retrospective nature of this study, we were not able to select doses of warfarin that are best suited for CURATE.AI dose optimisation subsequently, which would have influenced the dose recommendations coming from CURATE.AI. The sequential INR measurements in the dataset were not regularly spaced as well. Another limitation was that like‐for‐like comparison on technical performance metrics was unable to be performed between Curate.AI and the models selected from the literature because of the limited retrospective data set in this study and the lack of availability of raw data from these models in the literature. This in turn made certain statistical comparisons between CURATE.AI and these models not possible. Also, the newly devised metrics in this study have yet to be independently clinically validated. These new metrics were motivated by the lack of robust clinically relevant metrics in the literature to evaluate serial INR prediction performance. Our metrics were devised based on both clinical experience and literature review, making this study the first‐of‐its‐kind and suggesting these metrics for future application in studies on warfarin dosing algorithms.

CURATE.AI approach in itself is also not without limitations. The requirement for time and technical consistency between successive INR measurements may pose practical challenges. Before broad clinical deployment, CURATE.AI should be prospectively tested in an appropriately sized and controlled trial, with a study design that will not only answer fundamental questions of CURATE.AI technical performance, but also the pragmatic considerations of CURATE.AI's implementation into future patient care, toward improving personalized medicine in warfarin therapy and beyond.

## 5 CONCLUSIONS

After over 60 years of use, warfarin remains one of the most cost‐effective and widely prescribed medications for the prophylaxis and treatment of thromboembolic conditions. Still, warfarin management requires significant efforts in terms of frequent dose adjustments and can be challenging to use in the clinical setting. Fluctuations in INR can lead to adverse events including recurrent thromboembolism and life‐threatening hemorrhage. This results in significant medical and economic consequences and diminishes patient's quality of life.

CURATE.AI is a small data AI‐derived platform that aims to streamline the dose and drug optimisation in a personalized fashion. This retrospective study demonstrated that CURATE.AI may improve accuracy of warfarin dosing decisions compared to decisions made by unaided clinicians. CURATE.AI performed superiorly in terms of technical performance, illustrating CURATE.AI's robustness and supporting the potential application of CURATE.AI as a CDSS in the prospective management of warfarin dosing. The use of CURATE.AI in the clinical setting may facilitate the continued use of an established and cost‐effective drug like warfarin. This can have a significant impact both on the individual patient and public health.

## AUTHOR CONTRIBUTIONS

**Tiffany Rui Xuan Gan:** Conceptualization; methodology; data curation; investigation; writing – original draft; writing – review and editing; formal analysis. **Lester W. J. Tan:** Methodology; data curation; writing – original draft; formal analysis; writing – review and editing. **Mathias Egermark:** Writing – review and editing. **Anh T. L. Truong:** Methodology; formal analysis. **Kirthika Kumar:** Formal analysis. **Shi‐Bei Tan:** Formal analysis. **Sarah Tang:** Data curation. **Agata Blasiak:** Conceptualization; methodology; formal analysis; writing – review and editing. **Boon Cher Goh:** Conceptualization; writing – review and editing. **Kee Yuan Ngiam:** Conceptualization; writing – review and editing. **Dean Ho:** Conceptualization; writing – review and editing.

## CONFLICT OF INTEREST STATEMENT

Mathias Egermark is an employee of Roche Diagnostics and shareholder in F. Hoffmann‐La Roche Lester W. J. Tan, Anh T. L. Truong, Kirthika Kumar, Shi‐Bei Tan, Agata Blasiak and Dean Ho are co‐inventors of previously filed pending patents on artificial intelligence‐based therapy development. Dean Ho is a co‐founder and shareholder of KYAN Therapeutics, which has licensed intellectual property pertaining to AI‐based oncology drug development. The rest of the authors declare no conflict of interests.

## ETHICS STATEMENT

No animal or human subjects were involved in this study. Only retrospective data was collected for this study.

## Data Availability

Data sharing is not applicable to this article as no new data were created or analyzed in this study.

## References

[1] Johnson JA , Gong L , Whirl‐Carrillo M , et al. Clinical pharmacogenetics implementation consortium guidelines for CYP2C9 and VKORC1 genotypes and warfarin dosing. Clin Pharmacol Ther. 2011;90:625‐629. 21900891 10.1038/clpt.2011.185 PMC3187550

[2] Hamberg A‐K , Hellman J , Dahlberg J , Jonsson EN , Wadelius M . A Bayesian decision support tool for efficient dose individualization of warfarin in adults and children. BMC Med Inform Decis Mak. 2015;15:7. 25889768 10.1186/s12911-014-0128-0 PMC4324411

[3] Eriksson N , Wadelius M . Prediction of warfarin dose: why, when and how? Pharmacogenomics. 2012;13:429‐440. 22379999 10.2217/pgs.11.184

[4] Badjatiya A , Rao SV . Advances in antiplatelet and anticoagulant therapies for NSTE‐ACS. Curr Cardiol Rep. 2019;21:3. 30637536 10.1007/s11886-019-1090-3

[5] Patel S , Singh R , Preuss CV , Patel N . Warfarin. StatPearls. StatPearls Publishing; 2024. 29261922

[6] Doliner B , Jaller JA , Lopez AJ , Lev‐Tov H . Treatments to prevent primary venous ulceration after deep venous thrombosis. J Vasc Surg Venous Lymphat Disord. 2019;7:260‐271.e1. 30660582 10.1016/j.jvsv.2018.12.009

[7] Kimmel SE . Warfarin therapy: in need of improvement after all these years. Expert Opin Pharmacother. 2008;9:677‐686. 18345947 10.1517/14656566.9.5.677 PMC2855533

[8] Dalen JE , Alpert JS . Natural history of pulmonary embolism. Prog Cardiovasc Dis. 1975;17:259‐270. 1089991 10.1016/s0033-0620(75)80017-x

[9] Rosamond WD , Folsom AR , Chambless LE , et al. Stroke incidence and survival among middle‐aged adults: 9‐year follow‐up of the atherosclerosis risk in communities (ARIC) cohort. Stroke. 1999;30:736‐743. 10187871 10.1161/01.str.30.4.736

[10] Raskob GE , Angchaisuksiri P , Blanco AN , et al. Thrombosis: a major contributor to global disease burden. Arterioscler Thromb Vasc Biol. 2014;34:2363‐2371. 25304324 10.1161/ATVBAHA.114.304488

[11] di Nisio M , van Es N , Büller HR . Deep vein thrombosis and pulmonary embolism. Lancet. 2016;388:3060‐3073. 27375038 10.1016/S0140-6736(16)30514-1

[12] GBD 2019 Stroke Collaborators . Global, regional, and national burden of stroke and its risk factors, 1990‐2019: a systematic analysis for the global burden of disease study 2019. Lancet Neurol. 2021;20:795‐820. 34487721 10.1016/S1474-4422(21)00252-0 PMC8443449

[13] Wadelius M , Sörlin K , Wallerman O , et al. Warfarin sensitivity related to CYP2C9, CYP3A5, ABCB1 (MDR1) and other factors. Pharmacogenomics J. 2004;4:40‐48. 14676821 10.1038/sj.tpj.6500220

[14] Ageno W , Gallus AS , Wittkowsky A , et al. Oral anticoagulant therapy: antithrombotic therapy and prevention of thrombosis, 9th ed: American College of Chest Physicians Evidence‐Based Clinical Practice Guidelines. Chest. 2012;141:e44S‐e88S. 22315269 10.1378/chest.11-2292 PMC3278051

[15] Gallus AS , Baker RI , Chong BH , Ockelford PA , Street AM . Consensus guidelines for warfarin therapy. Recommendations from the Australasian Society of Thrombosis and Haemostasis. Med J Aust. 2000;172:600‐605. 10914107

[16] Sun S , Wang M , Su L , et al. Study on warfarin plasma concentration and its correlation with international normalized ratio. J Pharm Biomed Anal. 2006;42:218‐222. 16860509 10.1016/j.jpba.2006.03.019

[17] White CM . INR stability, clinical importance, and predictors in patients with atrial fibrillation and venous thromboembolism receiving vitamin K antagonists. Journal of Pharmacy Technology. 2016;32:253‐259.

[18] Kuruvilla M , Gurk‐Turner C . A review of warfarin dosing and monitoring. Proc (Bayl Univ Med Cent). 2001;14:305‐306. 16369639 10.1080/08998280.2001.11927781 PMC1305837

[19] Landefeld CS , Beyth RJ . Anticoagulant‐related bleeding: clinical epidemiology, prediction, and prevention. Am J Med. 1993;95:315‐328. 8368229 10.1016/0002-9343(93)90285-w

[20] Landefeld CS , Goldman L . Major bleeding in outpatients treated with warfarin: incidence and prediction by factors known at the start of outpatient therapy. Am J Med. 1989;87:144‐152. 2787958 10.1016/s0002-9343(89)80689-8

[21] Landefeld CS , Rosenblatt MW , Goldman L . Bleeding in outpatients treated with warfarin: relation to the prothrombin time and important remediable lesions. Am J Med. 1989;87:153‐159. 2757055 10.1016/s0002-9343(89)80690-4

[22] Lancaster TR , Singer DE , Sheehan MA , et al. The impact of long‐term warfarin therapy on quality of life. Evidence from a randomized trial. Boston area anticoagulation trial for atrial fibrillation investigators. Arch Intern Med. 1991;151:1944‐1949. 1929681

[23] Wadsworth D , Sullivan E , Jacky T , et al. A review of indications and comorbidities in which warfarin may be the preferred oral anticoagulant. J Clin Pharm Ther. 2021;46:560‐570. 33393699 10.1111/jcpt.13343

[24] Nishimura RA , Otto CM , Bonow RO , et al. 2017 AHA/ACC focused update of the 2014 AHA/ACC guideline for the Management of Patients with Valvular Heart Disease: a report of the American College of Cardiology/American Heart Association task force on clinical practice guidelines. Circulation. 2017;135:e1159‐e1195. 28298458 10.1161/CIR.0000000000000503

[25] Jawitz OK , Wang TY , Lopes RD , et al. Rationale and design of PROACT Xa: a randomized, multicenter, open‐label, clinical trial to evaluate the efficacy and safety of apixaban versus warfarin in patients with a mechanical on‐X aortic heart valve. Am Heart J. 2020;227:91‐99. 32693197 10.1016/j.ahj.2020.06.014 PMC7484170

[26] Aimo A , Giugliano RP , de Caterina R . Non‐vitamin K antagonist oral anticoagulants for mechanical heart valves: is the door still open? Circulation. 2018;138:1356‐1365. 30354416 10.1161/CIRCULATIONAHA.118.035612

[27] Eikelboom JW , Connolly SJ , Brueckmann M , et al. Dabigatran versus warfarin in patients with mechanical heart valves. N Engl J Med. 2013;369:1206‐1214. 23991661 10.1056/NEJMoa1300615

[28] Cohen D , Berger SP , Steup‐Beekman GM , Bloemenkamp KWM , Bajema IM . Diagnosis and management of the antiphospholipid syndrome. BMJ. 2010;340:c2541. 20472677 10.1136/bmj.c2541

[29] Cheung K‐S , Leung WK . Gastrointestinal bleeding in patients on novel oral anticoagulants: risk, prevention and management. World J Gastroenterol. 2017;23:1954‐1963. 28373761 10.3748/wjg.v23.i11.1954 PMC5360636

[30] Giugliano RP , Ruff CT , Braunwald E , et al. Edoxaban versus warfarin in patients with atrial fibrillation. N Engl J Med. 2013;369:2093‐2104. 24251359 10.1056/NEJMoa1310907

[31] Patel MR , Mahaffey KW , Garg J , et al. Rivaroxaban versus warfarin in nonvalvular atrial fibrillation. N Engl J Med. 2011;365:883‐891. 21830957 10.1056/NEJMoa1009638

[32] Wadhera RK , Russell CE , Piazza G . Cardiology patient page. Warfarin versus novel oral anticoagulants: how to choose? Circulation. 2014;130:e191‐e193. 25421049 10.1161/CIRCULATIONAHA.114.010426

[33] Heneghan CJ , Garcia‐Alamino JM , Spencer EA , et al. Self‐monitoring and self‐management of oral anticoagulation. Cochrane Database Syst Rev. 2016;7:CD003839. 27378324 10.1002/14651858.CD003839.pub3 PMC8078378

[34] Mujer MTP , Rai MP , Atti V , et al. An update on the reversal of non‐vitamin K antagonist oral anticoagulants. Adv Hematol. 2020;2020:7636104. 32231703 10.1155/2020/7636104 PMC7097770

[35] Xue L , Zhang Y , Xie C , et al. Relationship between warfarin dosage and international normalized ratio: a dose‐response analysis and evaluation based on multicenter data. Eur J Clin Pharmacol. 2019;75:785‐794. 31037455 10.1007/s00228-019-02655-8

[36] Ovesen L , Lyduch S , Ott P . A simple technique for predicting maintenance dosage of warfarin: is it better than empirical dosing? Eur J Clin Pharmacol. 1989;37:573‐576. 2612552 10.1007/BF00562547

[37] Asiimwe IG , Zhang EJ , Osanlou R , Jorgensen AL , Pirmohamed M . Warfarin dosing algorithms: a systematic review. Br J Clin Pharmacol. 2021;87:1717‐1729. 33080066 10.1111/bcp.14608 PMC8056736

[38] Wells PS , Holbrook AM , Crowther NR , Hirsh J . Interactions of warfarin with drugs and food. Ann Intern Med. 1994;121:676‐683. 7944078 10.7326/0003-4819-121-9-199411010-00009

[39] Jonas DE , McLeod HL . Genetic and clinical factors relating to warfarin dosing. Trends Pharmacol Sci. 2009;30:375‐386. 19540002 10.1016/j.tips.2009.05.001

[40] Truong ATL , Tan LWJ , Chew K , et al. Harnessing CURATE.AI for N‐of‐1 optimization analysis of combination therapy in hypertension patients: a retrospective case series (Adv. Therap. 10/2021). Adv Therap. 2021;4:2170030.

[41] Horwitz MA , Clemens DL , Lee B‐Y . AI‐enabled parabolic response surface approach identifies ultra short‐course near‐universal TB drug regimens. Adv Therap. 2020;3:1900086.

[42] Lee D‐K , Chang VY , Kee T , Ho C‐M , Ho D . Optimizing combination therapy for acute lymphoblastic leukemia using a phenotypic personalized medicine digital health platform: retrospective optimization individualizes patient regimens to maximize efficacy and safety. SLAS Technol. 2017;22:276‐288. 27920397 10.1177/2211068216681979

[43] Chakradhar S . Predictable response: finding optimal drugs and doses using artificial intelligence. Nat Med. 2017;23:1244‐1247. 29117178 10.1038/nm1117-1244

[44] Lee B‐Y , Clemens DL , Silva A , et al. Drug regimens identified and optimized by output‐driven platform markedly reduce tuberculosis treatment time. Nat Commun. 2017;8:14183. 28117835 10.1038/ncomms14183 PMC5287291

[45] Pantuck AJ , Lee D‐K , Kee T , et al. Modulating BET bromodomain inhibitor ZEN‐3694 and enzalutamide combination dosing in a metastatic prostate cancer patient using CURATE.AI, an artificial intelligence platform. Adv Therap. 2018;1:1800104.

[46] Tan BKJ , Teo CB , Tadeo X , et al. Personalised, rational, efficacy‐driven cancer drug dosing via an artificial intelligence SystEm (PRECISE): a protocol for the PRECISE CURATE.AI pilot clinical trial. Front Digit Health. 2021;3:635524. 34713106 10.3389/fdgth.2021.635524 PMC8521832

[47] Blasiak A , Kee TW , Rashid MBM , et al. Abstract CT268: CURATE.AI‐optimized modulation for multiple myeloma: an N‐of‐1 randomized trial. Cancer Res. 2020;80:CT268–CT268.

[48] Kee T , Weiyan C , Blasiak A , et al. Harnessing CURATE.AI as a digital therapeutics platform by identifying N‐of‐1 learning trajectory profiles. Adv Therap. 2019;2:1900023.

[49] Blasiak A , Khong J , Kee T . CURATE.AI: optimizing personalized medicine with artificial intelligence. SLAS Technol. 2020;25:95‐105. 31771394 10.1177/2472630319890316

[50] Zarrinpar A , Lee DK , Silva A , et al. Individualizing liver transplant immunosuppression using a phenotypic personalized medicine platform. Sci Transl Med. 2016;8:333ra49. 10.1126/scitranslmed.aac5954 27053773

[51] Vadher B , Patterson DL , Leaning M . Prediction of the international normalized ratio and maintenance dose during the initiation of warfarin therapy. Br J Clin Pharmacol. 1999;48:63‐70. 10383562 10.1046/j.1365-2125.1999.00967.x PMC2014877

[52] Ministry of Health Singapore . MOH Clinical Pharmacy Practice Guidelines. Anticoagulation ‐ Warfarin.

[53] McDonald S , Xydeas CS , Angelov PP . A retrospective comparative study of three data modelling techniques in anticoagulation therapy. 2008 International Conference on BioMedical Engineering and Informatics, vol. 1, 219–225. 2008.

[54] Xue L , Holford N , Ding X‐L , et al. Theory‐based pharmacokinetics and pharmacodynamics of S‐ and R‐warfarin and effects on international normalized ratio: influence of body size, composition and genotype in cardiac surgery patients. Br J Clin Pharmacol. 2017;83:823‐835. 27763679 10.1111/bcp.13157 PMC5346875

[55] Linardatos P , Papastefanopoulos V , Kotsiantis S . Explainable AI: a review of machine learning interpretability methods. Entropy (Basel). 2020;23. 10.3390/e23010018 PMC7824368 33375658

[56] Han C , Rundo L , Murao K , Nemoto T , Nakayama H . Bridging the gap between AI and healthcare sides: towards developing clinically relevant AI‐powered diagnosis systems. In: Maglogiannis I , Iliadis L , Pimenidis E , eds. Artificial Intelligence Applications and Innovations. Springer International Publishing; 2020:320‐333.

[57] Petty GW , Brown RD Jr , Whisnant JP , et al. Frequency of major complications of aspirin, warfarin, and intravenous heparin for secondary stroke prevention. A population‐based study. Ann Intern Med. 1999;130:14‐22. 9890845 10.7326/0003-4819-130-1-199901050-00004

[58] Gulløv AL , Koefoed BG , Petersen P . Bleeding during warfarin and aspirin therapy in patients with atrial fibrillation: the AFASAK 2 study. Atrial fibrillation aspirin and anticoagulation. Arch Intern Med. 1999;159:1322‐1328. 10386508 10.1001/archinte.159.12.1322

[59] Fihn SD , Callahan CM , Martin DC , et al. The risk for and severity of bleeding complications in elderly patients treated with warfarin. The National Consortium of anticoagulation clinics. Ann Intern Med. 1996;124:970‐979. 8624064 10.7326/0003-4819-124-11-199606010-00004

[60] Hylek EM , Regan S , Go AS , et al. Clinical predictors of prolonged delay in return of the international normalized ratio to within the therapeutic range after excessive anticoagulation with warfarin. Ann Intern Med. 2001;135:393‐400. 11560452 10.7326/0003-4819-135-6-200109180-00008

[61] Zareh M , Davis A , Henderson S . Reversal of warfarin‐induced hemorrhage in the emergency department. West J Emerg Med. 2011;12:386‐392. 22224125 10.5811/westjem.2011.3.2051 PMC3236169

[62] Garcia DA , Regan S , Henault LE , et al. Risk of thromboembolism with short‐term interruption of warfarin therapy. Arch Intern Med. 2008;168:63‐69. 18195197 10.1001/archinternmed.2007.23

[63] Kose E , Arai S , An T , et al. Analysis of factors affecting time in therapeutic range control after warfarin administration. Pharmazie. 2015;70(7):494‐498. 26373212

[64] Razouki Z , Ozonoff A , Zhao S , Jasuja GK , Rose AJ . Improving quality measurement for anticoagulation: adding international normalized ratio variability to percent time in therapeutic range. Circ Cardiovasc Qual Outcomes. 2014;7:664‐669. 25185245 10.1161/CIRCOUTCOMES.114.000804

[65] Rosendaal FR , Cannegieter SC , van der Meer FJ , Briët E . A method to determine the optimal intensity of oral anticoagulant therapy. Thromb Haemost. 1993;69:236‐239. 8470047

[66] Loeliger EA . Laboratory control, optimal therapeutic ranges and therapeutic quality control in oral anticoagulation. Acta Haematol. 1985;74:125‐131. 3938155 10.1159/000206187

[67] Yu H‐Y , Tsai H‐E , Chen Y‐S , Hung K‐Y . Comparison of warfarin dosage fluctuation with time in therapeutic range for bleeding or thromboembolism rate in Chinese patients. J Formos Med Assoc. 2019;118:611‐618. 30126761 10.1016/j.jfma.2018.07.021

[68] Connolly SJ , Pogue J , Eikelboom J , et al. Benefit of oral anticoagulant over antiplatelet therapy in atrial fibrillation depends on the quality of international normalized ratio control achieved by centers and countries as measured by time in therapeutic range. Circulation. 2008;118:2029‐2037. 18955670 10.1161/CIRCULATIONAHA.107.750000

[69] Morgan CL , McEwan P , Tukiendorf A , et al. Warfarin treatment in patients with atrial fibrillation: observing outcomes associated with varying levels of INR control. Thromb Res. 2009;124:37‐41. 19062079 10.1016/j.thromres.2008.09.016

[70] Okumura K , Komatsu T , Yamashita T , et al. Time in the therapeutic range during warfarin therapy in Japanese patients with non‐valvular atrial fibrillation: a multicenter study of its status and infuential factors. Circ J. 2011;75:2087‐2094. 21737950 10.1253/circj.cj-11-0350

[71] van Spall HGC , Wallentin L , Yusuf S , et al. Variation in warfarin dose adjustment practice is responsible for differences in the quality of anticoagulation control between centers and countries: an analysis of patients receiving warfarin in the randomized evaluation of long‐term anticoagulation therapy (RE‐LY) trial. Circulation. 2012;126:2309‐2316. 23027801 10.1161/CIRCULATIONAHA.112.101808

[72] Cotté F‐E , Benhaddi H , Duprat‐Lomon I , et al. Vitamin K antagonist treatment in patients with atrial fibrillation and time in therapeutic range in four European countries. Clin Ther. 2014;36:1160‐1168. 25151574 10.1016/j.clinthera.2014.07.016

[73] Singer DE , Hellkamp AS , Piccini JP , et al. Impact of global geographic region on time in therapeutic range on warfarin anticoagulant therapy: data from the ROCKET AF clinical trial. J Am Heart Assoc. 2013;2:e000067. 23525418 10.1161/JAHA.112.000067 PMC3603243

[74] Grzymala‐Lubanski B , Själander S , Renlund H , Svensson PJ , Själander A . Computer aided warfarin dosing in the Swedish national quality registry AuriculA: algorithmic suggestions are performing better than manually changed doses. Thromb Res. 2013;131:130‐134. 23232091 10.1016/j.thromres.2012.11.016

[75] Wieloch M , Själander A , Frykman V , et al. Anticoagulation control in Sweden: reports of time in therapeutic range, major bleeding, and thrombo‐embolic complications from the national quality registry AuriculA. Eur Heart J. 2011;32:2282‐2289. 21616951 10.1093/eurheartj/ehr134

[76] Heneghan C , Ward A , Perera R , et al. Self‐monitoring of oral anticoagulation: systematic review and meta‐analysis of individual patient data. Lancet. 2012;379:322‐334. 22137798 10.1016/S0140-6736(11)61294-4

